# KP772 overcomes multiple drug resistance in malignant lymphoma and leukemia cells in vitro by inducing Bcl-2-independent apoptosis and upregulation of Harakiri

**DOI:** 10.1007/s00775-021-01900-9

**Published:** 2021-10-06

**Authors:** Lisa Kater, Benjamin Kater, Michael A. Jakupec, Bernhard K. Keppler, Aram Prokop

**Affiliations:** 1 Department of Pediatric Oncology/Hematology, University Medical Center Charité, Campus Virchow, Augustenburger Platz 1, 13353 Berlin, Germany; 2 Present Address: Department of Naturopathy, Immanuel Hospital Berlin, Königstraße 63, 14109 Berlin, Germany; 3 Present Address: MVZ Nuclear Medicine, Vivantes Hospital “Am Urban”, Dieffenbachstraße 1, 10967 Berlin, Germany; 4 Faculty of Chemistry, Institute of Inorganic Chemistry, University of Vienna, Währinger Straße 42, 1090 Vienna, Austria; 5 Research Cluster “Translational Cancer Therapy Research”, University of Vienna, Währinger Straße 42, 1090 Vienna, Austria; 6 Present Address: Department of Pediatric Hematology/Oncology, Helios Clinic Schwerin, Wismarsche Straße 393–397, 19049 Schwerin, Germany; 7 Present Address: Department of Pediatric Hematology/Oncology, Children’s Hospital Cologne, Amsterdamerstraße 59, 50735 Cologne, Germany; 8 Medical School Hamburg (MSH), University of Applied Sciences and Medical University, Am Kaiserkai 1, 20457 Hamburg, Germany

**Keywords:** Lanthanum, Apoptosis, Multidrug resistance, Bcl-2, Harakiri

## Abstract

Despite high cure rates in pediatric patients with acute leukemia, development of resistance limits the efficacy of antileukemic therapy. *Tris*(1,10-phenanthroline)*tris*(thiocyanato-κ*N*)lanthanum(III) (KP772) is an experimental antineoplastic agent to which multidrug-resistant cell models have shown hypersensitivity. Antiproliferative and apoptotic activities of KP772 were tested in leukemia, lymphoma and solid tumor cell lines as well as primary leukemia cells (isolated from the bone marrow of a child with acute myeloid leukemia (AML). The ability to overcome drug resistances was investigated in doxorubicin- and vincristine-resistant cell lines. Real-time PCR was used to gain insight into the mechanism of apoptosis induction. KP772 inhibited proliferation and induced apoptosis in various leukemia and lymphoma cell lines in a concentration-dependent manner (LC_50_ = 1–2.5 µM). Primary AML cells were also sensitive to KP772, whereas daunorubicin showed no significant effect. KP772 induces apoptosis independently of Bcl-2, Smac, and the CD95 receptor and is also effective in caspase 3-deficient MCF7 cells, indicating that apoptosis is partly triggered independently of caspase 3. mRNA expression profiling revealed an upregulation of the BH3-only Bcl-2 protein Harakiri in the course of KP772-induced apoptosis. Remarkably, KP772 overcame drug resistance to doxorubicin and vincristine in vitro, and the apoptotic effect in resistant cells was even superior to that in non-resistant parental cells. In combination with vincristine, doxorubicin and cytarabine, synergistic effects were observed in BJAB cells. The cytotoxic potency in vitro/ex vivo and the remarkable ability to overcome multidrug resistance propose KP772 as a promising candidate drug for antileukemic therapy, especially of drug-refractory malignancies.

**Graphic abstract**

**Supplementary Information:**

The online version contains supplementary material available at 10.1007/s00775-021-01900-9.

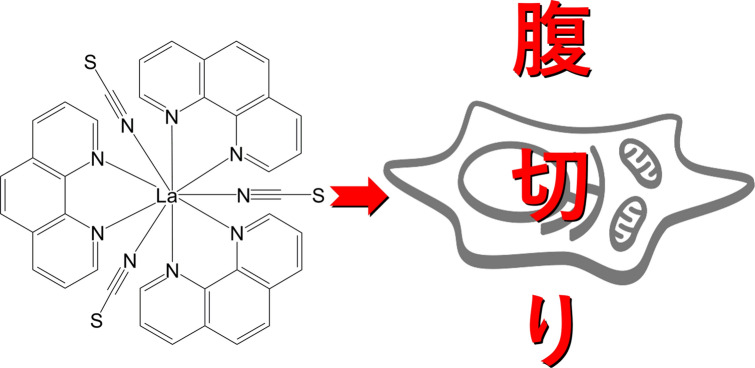

## Introduction

**Fig. 1:**
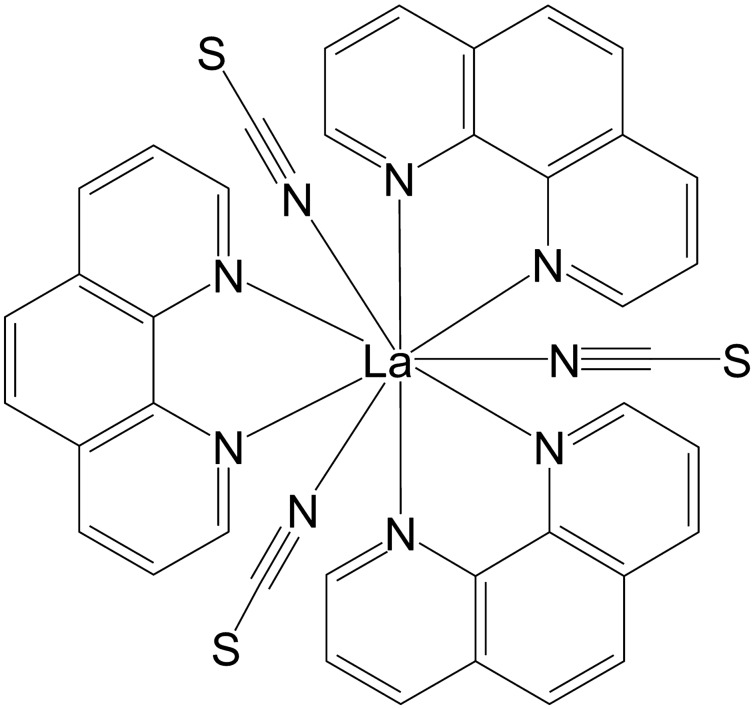
Structural formula of tris(1,10-phenanthroline)tris(thiocyanato-κ*N*)lanthanum(III) (KP772)

In recent decades, great efforts were made to improve the outcome of patients with acute leukemia. Nowadays, most children suffering from acute leukemia can be cured by standardized combination chemotherapies. Nevertheless, the development of drug resistances limits the efficiency of therapy. In quest of new drug candidates, we examined the lanthanum complex *tris*(1,10-phenanthroline)*tris*(thiocyanato-κ*N*)lanthanum(III) (KP772; Fig. 1) for its cytotoxicity with emphasis on hematological malignancies, its ability to overcome multiple drug resistances, and the apoptotic signaling pathways involved.

Unlike necrosis, apoptosis is a form of programmed cell death, which is essential in various biological settings, such as different levels of embryonic development, tissue homeostasis, development of the nervous system and the immune system, and in chemically induced cell death [1]. Two main pathways lead to apoptosis, the extrinsic (death receptor-mediated) and the intrinsic (mitochondrial) pathway. Defects in apoptotic pathways are involved in tumorigenesis, and inactivation of apoptotic signaling is central in the development of drug resistance [1].

Genes involved in regulation of apoptosis include p53 and various pro- and anti-apoptotic members of the Bcl-2 (B-cell lymphoma 2 protein) family. P53-mediated apoptosis implies upregulation of Bax (Bcl-2-associated X protein) and downregulation of Bcl-2, both important regulators of apoptosis [2, 3]. However, upregulation of Bcl-2 is common in acute leukemia and a decreased Bax/Bcl-2 ratio correlates with poor response to therapy [1]. Another important factor for drug resistance is the expression of ATP-binding cassette (ABC) transporters leading to increased efflux and decreased intracellular accumulation of various anticancer agents [4, 5].

KP772 has shown cytotoxicity in the low micromolar range in numerous human tumor cell lines in vitro, with two leukemia cell lines being among the most sensitive in the panel of the National Cancer Institute (USA). Apart from strong anti-invasive properties, its cytotoxicity remained unaffected in multicellular tumor spheroids (in contrast to several established or investigational metal-based anticancer drugs), suggesting both a high capacity of penetrating multiple cell layers and a low dependency on cell cycle activity [6]. Therapeutic efficacy in vivo has been demonstrated in a DLD-1 colon carcinoma xenograft model [7]. KP772 exerts anticancer effects by G_0_/G_1_ cell cycle arrest, probably unrelated to activation of p53, but possibly involving interference with iron homeostasis [8], and by apoptosis via the mitochondrial pathway, which is not caused by DNA damage or radical-mediated damage [7]. Although DNA intercalation and DNA groove binding have been suggested as modes of action for (remotely) related complexes with phenanthroline derivatives [9–12], they seem to be irrelevant for KP772 [7]. Likewise, photo-induced cytotoxicity as a basis for application in photodynamic therapy has been pursued in the case of a lanthanum complex with photo-active phenanthroline derivatives (dipyridophenazine), but not the parent complex with phenanthroline [12].

Multidrug-resistant cells overexpressing ABC transporters such as ABCB1 (P-glycoprotein, P-gp), ABCC1 (multidrug resistance protein 1), or ABCG2 (breast cancer resistance protein) were found to be hypersensitive to KP772. Even more remarkably, continuous exposure of ABCB1-overexpressing cells to subcytotoxic concentrations of KP772 restored their sensitivity to anticancer drugs such as vincristine or doxorubicin, probably not involving direct interaction between KP772 and the transporter but rather a reduction of ABCB1 expression [13].

The present study aimed to extend our knowledge on the ability of KP772 to overcome both P-gp-mediated and P-gp-independent drug resistances. Furthermore, the impact on mRNA expression of 84 apoptosis-relevant genes was analyzed. To provide a basis for future drug combination studies, synergies with established cytostatic drugs (doxorubicin, vincristine, cytarabine) were explored in lymphoma cells.

## Materials and methods

### Materials

RNase A was from Qiagen (Hilden, Germany), propidium iodide (50 µg/ml) from Serva (Heidelberg, Germany), and doxorubicine (Doxo), vincristine (Vcr) and cytarabine (AraC) were provided by the Charité, Berlin, Germany. Drugs were freshly dissolved in dimethylsulfoxide (DMSO) prior to the experiments and diluted with the appropriate medium or buffer during the assay procedures. *Tris*(1,10-phenanthroline)*tris*(thiocyanato-κ*N*)lanthanum(III) (KP772) was prepared at the Institute of Inorganic Chemistry, University of Vienna, as described previously [14]. For each experiment, KP772 was freshly dissolved in 0.4% NaCl solution to give a 1 mM stock solution.

### Cell lines and cell culture

The cell line BJAB (human Burkitt-like lymphoma) and the construct BJAB mock/FADD-dn were used. BJAB FADD-dn cells, stably transfected with a dominant-negative FADD (Fas-associated protein with death domain) mutant lacking the N-terminal death effector domain (pcDNA3-FADD-dn-transfected), were provided by Peter Daniel, Charité, Berlin. The FADD-dn mutant completely blocks CD95-mediated apoptosis [15]. Doxorubicin-resistant BJAB cells (7CCA) were generated by exposing BJAB cells to increasing doxorubicin concentrations of up to 1 µg/ml. Nalm-6 cells (human B-cell precursor leukemia) were provided by AG Henze, Charité, Berlin. To generate a vincristine-resistant Nalm-6 cell line (Nalm-6/Vcr), Nalm-6 cells were exposed to increasing concentrations of vincristine up to 30 nM.

The cell construct Jurkat (human T-cell leukemia) neo/Smac, where the Smac line over-expresses the pro-apoptotic Smac protein, was provided by PD Dr. Fulda, University Ulm. The construct MelHO (human melanoma) pIres/Bcl-2, provided by Dr. Eberle, Charité, Berlin, consists of MelHO cells stably transfected with the pIres (plasmid with internal ribosome entry site sequence) vector and pIres-Bcl-2, the latter strongly over-expressing the anti-apoptotic Bcl-2 protein. MCF7 (human breast adenocarcinoma) cells were obtained from Deutsche Sammlung von Mikroorganismen und Zellkulturen (DSMZ).

Cell lines were maintained in 250-ml cell culture flasks at 37 °C. Suspension cells were grown in RPMI 1640 medium (Gibco, Invitrogen, Karlsruhe, Germany) supplemented with heat-inactivated fetal calf serum (FCS, 10%, v/v), L-glutamine (0.56 g/l), penicillin (100,000 i.u.) and streptomycin (0.1 g/l). Adherent cells were grown in Dulbecco’s modified minimal essential medium (DMEM) supplemented with FCS (10%, v/v) and geniticine (0.4 g/l). Cells were passaged 2–3 times per week by dilution to 1 × 10^5^ cells/ml. 24 h before the assay setup, cells were adjusted to 3 × 10^5^ cells/ml to ascertain standardized growth conditions. For proliferation and apoptosis assays, cells were diluted to 1 × 10^5^ cells/ml immediately before treatment.

### Primary cells

Primary leukemia cells from a child with acute myeloblastic leukemia (AML) were obtained by bone marrow aspiration. Diagnosis was established by immunophenotyping of leukemia cells according to Béné et al. [16]. Primary leukocytes from a healthy person were obtained from peripheral blood. Lymphoblasts and mononuclear cells were separated by centrifugation over a Ficoll gradient. After separation, the percentage of leukemia cells was higher than 95%, in accordance with a former study [1]. Primary cells were immediately seeded at a density of 3 × 10^5^ cells/ml in RPMI 1640 complete cell culture medium in 6-well plates and incubated for 60 h with different agents for the apoptosis assays. Levels of DNA fragmentation were measured already after 60 h because of high unspecific cell death observed in control cells after longer periods.

### Measurement of non-apoptotic cell death by LDH-release assay

Unspecific cytotoxicity was assessed by measuring the release of lactate dehydrogenase (LDH) from BJAB cells (treated with KP772 for 2 h) with the Cytotoxicity Detection Kit from Boehringer Mannheim (Mannheim, Germany). Supernatants were centrifuged at 1500 rpm for 5 min. 20 µl of cell-free supernatants were diluted with 80 µl phosphate-buffered saline (PBS), and 100 µl reaction mixture containing 2-(4-iodophenyl)-3-(4-nitrophenyl)-5-phenyl-2H-tetrazolium chloride (INT), sodium lactate, NAD^+^ and diaphorase were added. Time-dependent formation of the reaction product was quantified photometrically at 490 nm. LDH activity released by cells lyzed with 0.1% Triton X-100 in culture medium was taken as 100%.

### Determination of cell density and cell viability

Cell count and viability were measured with a CASY^®^ Cell Counter and Analyzer System (Innovatis, Bielefeld, Germany) as described in literature [17, 18]. Parameters measured were adjusted to the requirements of the cells used. With this system, cell density can be analyzed simultaneously in different size ranges: cell debris, dead cells, and viable cells. Cells were seeded at a density of 1 × 10^5^ cells/ml in 6-well plates and treated with the respective agent in comparison to untreated and DMSO controls. After 24 h incubation at 37 °C, cells were resuspended properly and 100 µl from each well were diluted in CASYton (ready-to-use isotonic saline solution, 10 ml) for immediate cell counting. The frequency of cells in untreated controls was defined as 100% growth. Maximal inhibition of proliferation was achieved when the cell density was not higher than at the beginning of the experiment.

### Measurement of apoptosis

DNA fragmentation during the late phase of apoptosis was measured by a modified cell cycle analysis as described in literature [19, 20]. After incubation for 72 h or 60 h at 37 °C in 6-well plates, cells were collected by centrifugation at 1500 rpm for 5 min, washed with PBS at 4 °C and fixed in PBS/2% (v/v) formaldehyde on ice for 30 min. After fixation, cells were pelleted, incubated with ethanol/PBS (2:1, v/v) for 15 min, pelleted and resuspended in PBS containing RNase A (40 µg/ml). RNA was digested for 30 min at 37 °C; cells were pelleted once again and resuspended in PBS containing propidium iodide (50 µg/ml). Nuclear DNA fragmentation was quantified by flow cytometric determination of hypodiploid DNA. Data were collected and analyzed with a FACScan (Becton Dickinson, Heidelberg, Germany) instrument equipped with CellQuest software. Percentages of hypoploidy (subG1) reflect the number of apoptotic cells. Background apoptosis (in control cells) was subtracted from total apoptosis in treated cells. Cell death in the early phase of apoptosis was determined using the Annexin V-propidium iodide binding assay by double-staining cell lines with annexin-V–FITC (fluorescein isothiocyanate) and propidium iodide (PI), as described previously [21]. The cell line Nalm-6 as well as primary leukocytes of a healthy person were used for this experiment. A total of 1 × 10^5^ cells were washed twice with ice-cold PBS and then resuspended in binding buffer containing 4-(2-hydroxyethyl)-1-piperazineethanesulfonic acid (HEPES, 10 mM)/NaOH (pH 7.4), NaCl (140 mM) and CaCl_2_ (2.5 mM). Next, annexin-V–FITC (10 µl) from BD Pharmingen (Heidelberg, Germany) and PI (10 μl, 50 μg/ml) from Sigma-Aldrich (Taufkirchen, Germany) were added to the cells. Analyses were performed after 48 h on a FACScan instrument (Becton Dickinson, Heidelberg, Germany), using the CellQuest analysis software. The amount of cells which were both annexin-V-FITC-negative and PI-negative after 48 h were classified as viable, the other cells were classified as undergoing cell death.

### Gene expression analysis

For analysis of the differential expression of multiple genes involved in apoptosis pathways, we used the apoptosis-specific RT2 profiler PCR expression array (SuperArray PAHS-012; SABiosciences Corporation, Frederick, MD, USA) comprising 84 genes involved in apoptosis signal cascades according to the manufacturer’s instructions. Total RNA was extracted from BJAB cells incubated with KP772 for 8 h, and RNAs were treated with DNase I (2 U/µl) to eliminate possible genomic DNA contamination. Total RNA (700 ng/µl) was then used as a template for synthesis of a cDNA probe and subjected to quantitative real-time PCR SuperArray analysis using a LightCycler 480 (Roche Diagnostics). Means of nine housekeeping genes were used to normalize hybridization signals. Results were analyzed using SuperArray software, and expression of the respective genes is given relative to control cells incubated in vehicle-containing medium for 8 h.

### Statistical analysis

The data in the diagrams are shown as mean values from three independent samples of one approach, and standard deviations are given by the error indicators. Data evaluation and statistical calculations were carried out with Microsoft Excel. The evaluation of the flow-cytometric measurements was carried out with the CellQuest Pro software. The AUC, which reflects the proportion of the hypodiploid sub-G1 population, was determined in the histograms of the modified cell cycle analysis. In the case of the dot plots of the annexin V / PI double staining, the distributions of the cells in the quadrants were determined and used for the calculations. The significance of differences in the comparison of data was calculated using the two-sided Student`s *t* test, with *p* < 0.05 rated as statistically significant) (*), *p* < 0.01 as highly significant (**) and *p* < 0.001 as extremely significant (***); *p* > 0.05 was rated as not significant (n.s.). To determine synergistic effects, the fractional product concept was applied, which is a widely used method for analyzing synergism, antagonism and summation of effects [22]. The fractional product was calculated according to the following formula: *Fp* = *E*1; 2/(*E*1 + *E*s-*E*1 × *E*2). *Fp* < 1 indicates an antagonistic effect, *Fp* = 1 an additive effect and *Fp* > 1 a synergistic effect [23].

## Results

### Exclusion of unspecific cytotoxicity

Unspecific cytotoxic effects of KP772 could be excluded by determination of extracellular LDH release by ELISA (enzyme-linked immunosorbent assay) detection. As shown in Fig. S1, KP772 did not result in leakage of LDH from BJAB cells after incubation for 2 h, indicating that a necrosis-like mechanism is not responsible for the activity of KP772.

### Antiproliferative effects of KP772

**Fig. 2:**
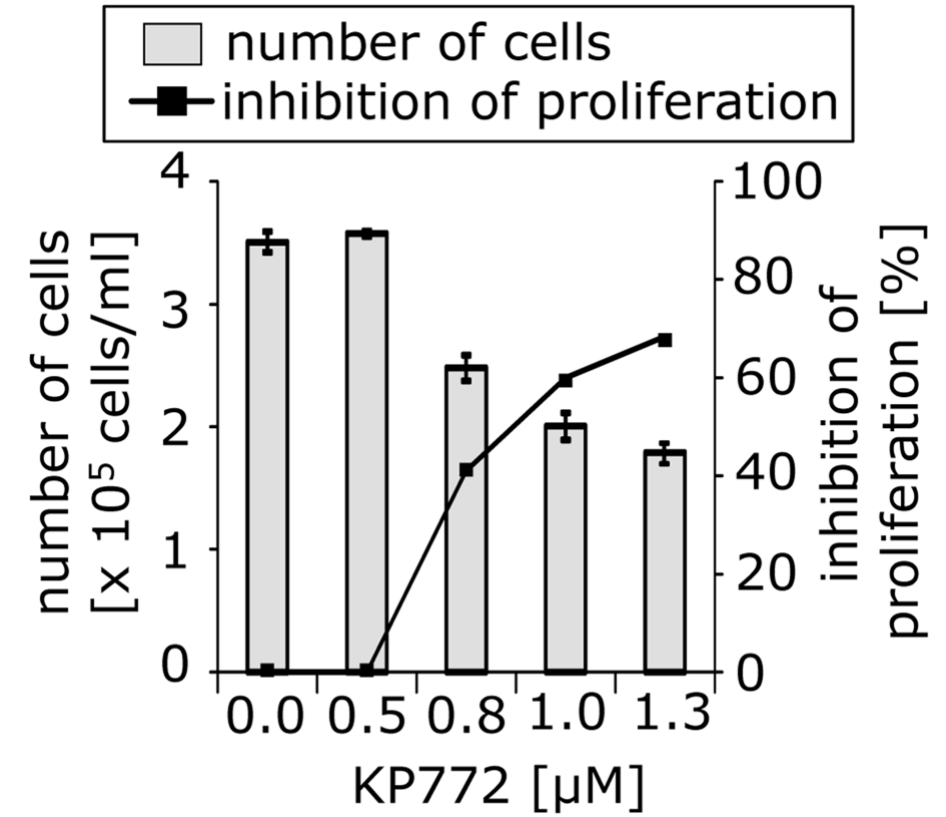
KP772 inhibits cell proliferation in BJAB cells. Cells were either left untreated as control or incubated with different concentrations of KP772. After 24 h, the cell proliferation was determined using the CASY® Cell Counter + Analyzer System. Inhibition of proliferation is given in % of control ± SD (*n* = 3)

To investigate the antiproliferative activity of KP772, we measured viability and cell count of various lymphoma and leukemia cell lines, such as BJAB, NALM-6 and Jurkat, after incubation for 24 h with different concentrations of the agent. Cell debris, dead cells and viable cells were determined in a single measurement with settings specifically defined for the cells used. KP772 decreases tumor cell proliferation in a concentration-dependent manner (Figs. S2–S6). Inhibition appeared at a concentration of 0.6 µM, was 40% at 0.8 µM, and the effect increased to 70% inhibition at 1.2 µM in BJAB cells (Fig. 2).

### *KP772 induces apoptosis *in vitro* and *ex vivo

**Fig. 3:**
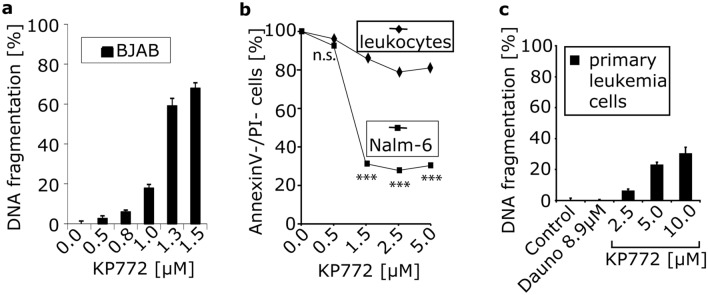
**a** KP772 induces DNA fragmentation in BJAB cells. Cells were either left untreated as control or incubated with different concentrations of KP772. After 72 h, DNA fragmentation was measured by flow-cytometric analysis. Values of DNA fragmentation are given in % ± SD (*n* = 3); **b** Nalm-6 cells and primary leukocytes obtained from a healthy person were either left untreated as controls or incubated with different concentrations of KP772. The percentage of viable cells (annexin-V-/PI-) was measured after 48 h. The asterisks indicate the significance of the differences in the number of viable cells between primary leukocytes and the leukemia cell line (***p < 0.001; n.s. not significant); **c** Primary tumor cells from a patient with AML were either incubated with daunorubicin or with different concentrations of KP772. Daunorubicin was used at 8.9 µM, which is the appropriate LC_50_ value, as determined by the annexin V/PI binding assay in BJAB cells. Some cells were left untreated as controls. DNA fragmentation was measured after 60 h by flow-cytometric analysis. Values are given in % ± SD (*n* = 3)

Apoptotic cells undergo characteristic morphological changes, such as cell shrinkage, coalescence and margination of chromatin, fragmentation of the cell and the nucleus. After incubation with 1 µM KP772 for 72 h, treated cells showed several apoptotic features. To quantify induction of apoptosis, we determined DNA fragmentation (an accepted hallmark of apoptosis) by flow cytometric measurement of hypodiploid DNA. KP772 very effectively induced apoptosis in leukemia and lymphoma cell lines, such as BJAB (Fig. 3a), Jurkat and NALM-6, and even in multiresistant cell lines from solid tumors, such as MelHO (melanoma) and MCF7 (breast cancer).

To examine the specificity of the cytotoxic effect, we exposed NALM-6 cells and healthy leukocytes to the agent. KP772 induced apoptosis particularly in malignant cells, whereas healthy leukocytes were far less strongly affected (Fig. 3b). To investigate whether KP772 is capable of inducing apoptosis in leukemic cells ex vivo, primary leukemic cells isolated from a bone marrow aspiration of a child with AML were treated with up to 10 µM KP772, and DNA fragmentation was measured after 60 h. While the cells responded poorly to daunorubicin, KP772 induced apoptosis efficiently in up to 30% of cells (Fig. 3c).

### KP772-induced apoptosis is independent of the CD95 receptor and partly independent of caspase 3

**Fig. 4:**
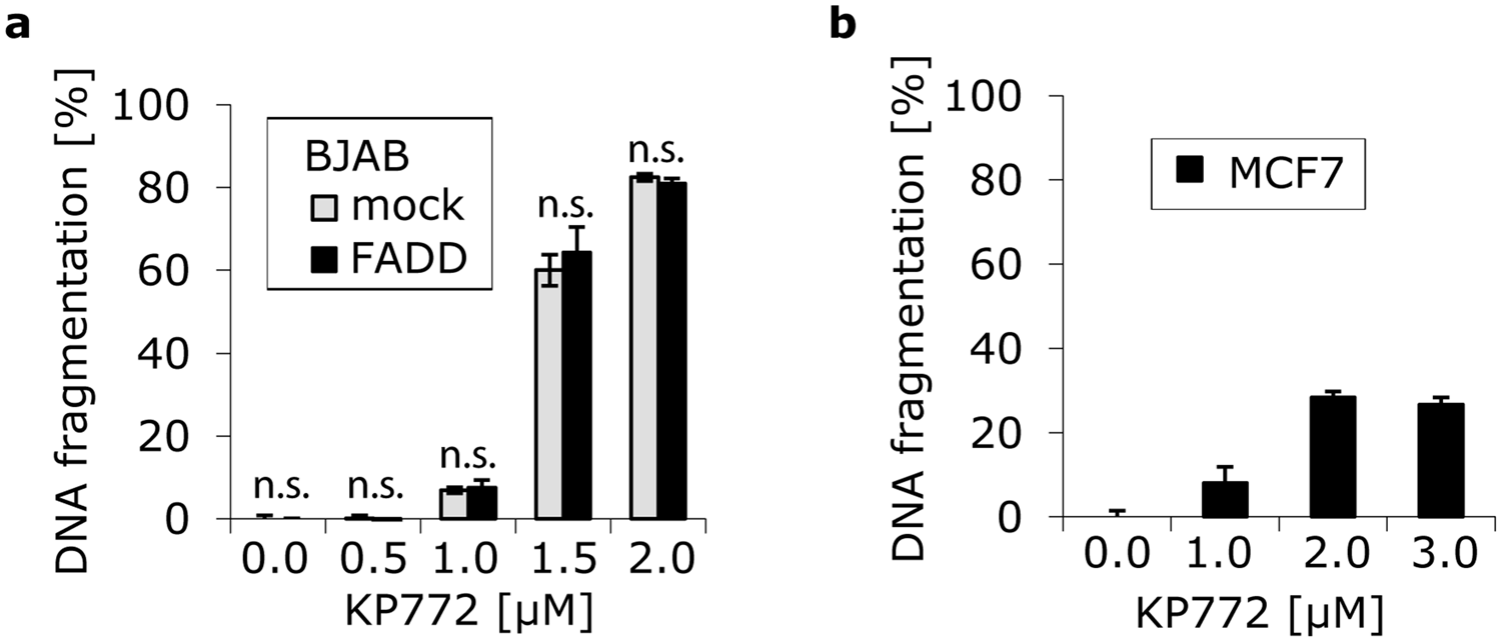
**a** BJAB FADD-dn cells (overexpressing a dominant negative FADD mutant) and BJAB mock cells (expressing endogenous FADD only) were either incubated with KP772 at different concentrations or left untreated as control. After 72 h, DNA fragmentation was measured by flow-cytometric analysis. Values of DNA fragmentation are given in % ± SD (*n* = 3). The differences in DNA fragmentation between mock and FADD-dn cell lines turned out to be insignificant after calculation of the *p* values and were marked accordingly (n.s.); **b** MCF7 cells were either incubated with KP772 at different concentrations or left untreated as control. After 72 h, DNA fragmentation was measured by flow-cytometric analysis. Values of DNA fragmentation are given in % ± SD (*n* = 3)

A model system consisting of BJAB mock and FADD-dn cells (the latter overexpressing a dominant-negative FADD mutant) was used to investigate whether the CD95 receptor takes part in KP772-induced cell death. The observation that FADD-dn- and mock-transfected cells show a comparable extent of DNA fragmentation (Fig. 4a) indicates that KP772-induced apoptosis is independent of the CD95 receptor. To further delineate the apoptotic pathway involved, we incubated caspase-3-deficient MCF7 breast cancer cells with KP772 (Fig. 4b). Results indicate that KP772 in low micromolar concentrations triggers apoptosis partly independent of caspase-3.

### KP772-induced apoptosis is independent of Smac and Bcl-2 and associated with upregulation of Harakiri

**Fig. 5:**
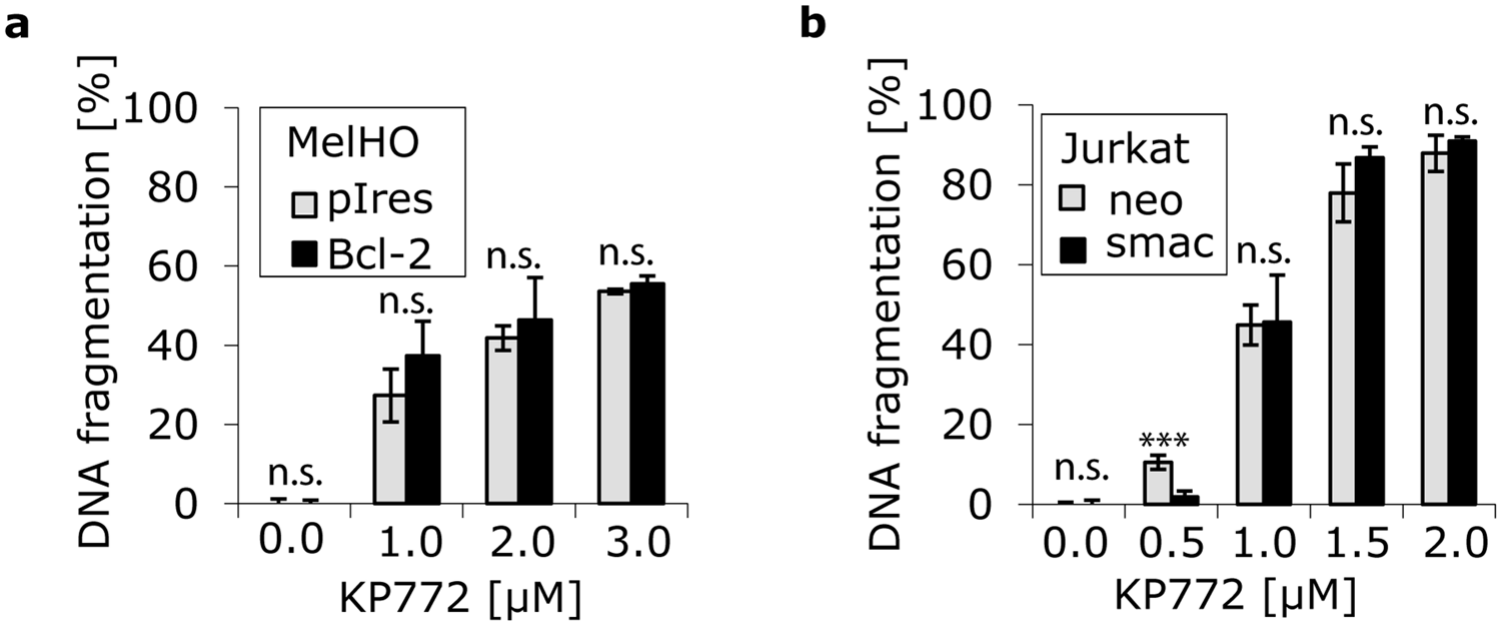
**a** MelHO Bcl-2 cells (overexpressing anti-apoptotic Bcl-2 protein) and MelHO pIres cells were either incubated with KP772 at different concentrations or left untreated as control. After 72 h, DNA fragmentation was measured by flow cytometric analysis. Values of DNA fragmentation are given in % ± SD (*n* = 3). The differences in DNA fragmentation between Bcl-2 and pIres cell lines were not significant after calculation of the *p* values and were marked accordingly (n.s.); **b** Jurkat smac cells (overexpressing the pro-apoptotic SMAC protein) and Jurkat neo cells were either incubated with KP772 at different concentrations or left untreated as control. After 72 h, DNA fragmentation was measured by flow-cytometric analysis. Values of DNA fragmentation are given in % ± SD (*n* = 3). As there were mostly insignificant differences in DNA fragmentation between smac and neo cell lines after calculation of the *p* values, they were marked with n.s.

To obtain further insights into the apoptotic pathways, we used cellular model systems with specific deficiencies in apoptosis cascades. Comparison of a transfected MelHO melanoma subline overexpressing Bcl-2 and a Jurkat T-cell leukemia subline overexpressing Smac with the respective vector-only controls showed no differences in the extent of DNA fragmentation upon treatment with KP772, suggesting that KP772-induced cell death is independent of the anti-apoptotic protein Bcl-2 and the pro-apoptotic factor Smac (Fig. 5). By real-time PCR, we investigated the mRNA expression of 84 apoptosis-relevant genes. 15 h after treatment with KP772, a fivefold upregulation of the gene encoding for the Bcl-2-interacting BH3(Bcl-2 homology domain 3)-only protein Harakiri was detected, while expression of all other analyzed genes was altered insignificantly (less than twofold).

### KP772 overcomes resistances to vincristine and doxorubicin

**Fig. 6:**
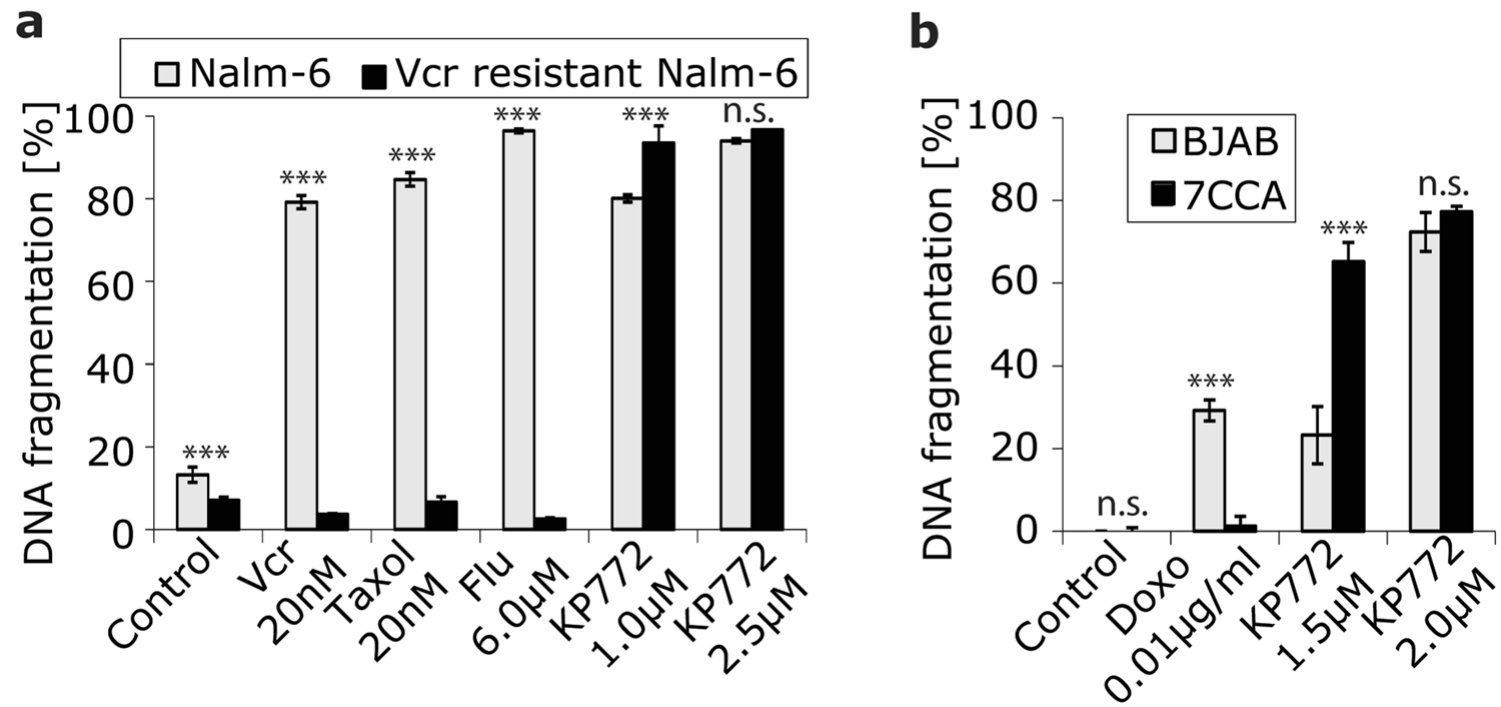
**a** Vincristine-resistant Nalm-6 cells (Nalm-6/Vcr) and the corresponding nonresistant cells (Nalm-6) were incubated with KP772, vincristine, paclitaxel and fludarabine. Vincristine was applied at 20 nM, paclitaxel and fludarabine were used at 20 nM and 6 µM, respectively. Some cells were left untreated as controls. DNA fragmentation was measured after 72 h by flow-cytometric analysis. Values of DNA fragmentation are given in % ± SD (*n* = 3). The asterisks mark the significance of the differences in induced DNA fragmentation between the resistant and non-resistant cell line; **b** Doxorubicin-resistant BJAB cells (7CCA) and the corresponding nonresistant cells (BJAB) were incubated with KP772 and doxorubicin. Doxorubicin was applied at 0.01 µg/ml. Some cells were left untreated as controls. DNA fragmentation was measured after 72 h by flow-cytometric analysis. Values of DNA fragmentation are given in % ± SD (*n* = 3). The asterisks mark the significance of the differences in induced DNA fragmentation between the resistant and non-resistant cell line (***p < 0.001; n.s. not significant)

Classic multidrug resistance (MDR) arises from P-gp-dependent cellular export of structurally diverse substances such as anthracyclines and *Vinca* alkaloids [24]. Thereby, cells become resistant to several other drugs in addition to the compound to which they have been exposed [25]. Flow cytometric analysis revealed a significant upregulation of P-gp in vincristine-resistant NALM-6 cells, consistent with their co-resistance to paclitaxel and fludarabine. In contrast, no upregulation of P-gp was detected in specifically doxorubicin-resistant BJAB cells (7CCA). The capacity of KP772 to overcome drug resistances was investigated in both cell models, revealing that KP772 not only overcomes these resistances, but is even more effective in the resistant cells than in the non-resistant parental cells (Fig. 6).

### KP772 shows synergistic effects with doxorubicin, vincristine and cytarabine

**Fig. 7:**
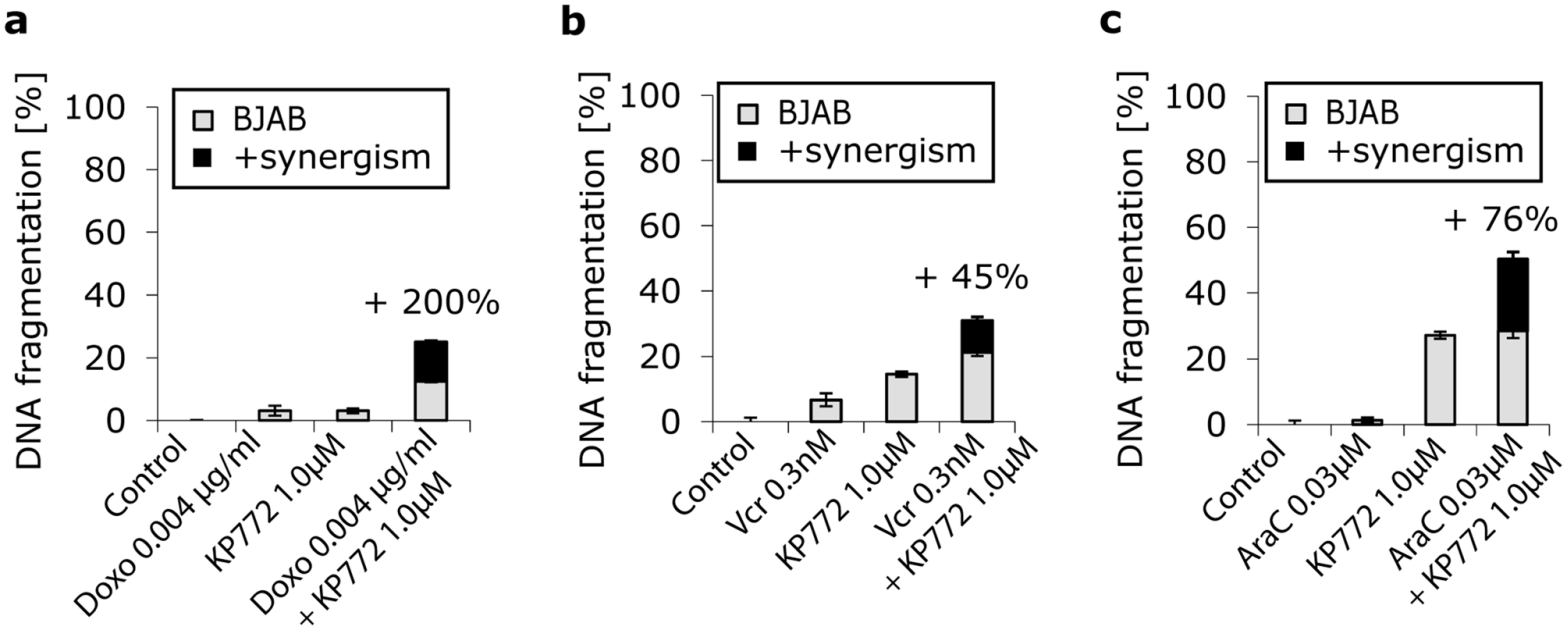
**a** BJAB cells were either incubated with KP772 or with doxorubicin (0.004 µg/ml) or with combinations of both compounds. The agents were applied at very low concentrations to investigate possible synergistic effects. Control cells were left untreated. After 72 h, DNA fragmentation was measured by flow cytometric analysis. Values of DNA fragmentation are given in % ± SD (*n* = 3); **b** BJAB cells were either incubated with KP772 or with vincristine (0.3 nM) or with combinations of both compounds. The agents were applied at very low concentrations to investigate possible synergistic effects. Control cells were left untreated. After 72 h, DNA fragmentation was measured by flow-cytometric analysis. Values of DNA fragmentation are given in % ± SD (*n* = 3); **c** BJAB cells were either incubated with KP772 or with cytarabine (0.03 µM) or with combinations of both compounds. The agents were applied at very low concentrations to investigate possible synergistic effects. Control cells were left untreated. After 72 h, DNA fragmentation was measured by flow-cytometric analysis. Values of DNA fragmentation are given in % ± SD (*n* = 3)

To investigate synergism with conventional cytostatic drugs, BJAB cells were incubated with KP772 plus one of the following: the anthracycline doxorubicin, the *Vinca* alkaloid vincristine, and the antimetabolite cytarabine. Significant synergistic interactions between KP772 and each of these drugs were observed with regard to apoptosis induction (Fig. 7).

## Discussion

In the present study, we investigated the ability of the lanthanum complex KP772 to initiate apoptosis in tumor cells and to overcome resistances to conventional cytostatic drugs in a wide range of cell lines from leukemia, lymphomas and solid tumors. Since tumor cells typically show high rates of cell proliferation, blocking the quantitative increase of malignant cells is an obvious goal of cancer treatment. However, eventually, curative treatment requires a definite elimination of tumor cells by apoptotic cell death. KP772 turned out to be a strong inhibitor of tumor cell proliferation (by up to 70% after 24 h), but moreover, KP772 effectively induces apoptosis in each cell line examined at low concentrations (LC_50_ = 1–2.5 µM).

Apoptosis is a form of programmed cell death which involves several biochemical events leading to characteristic changes in cell morphology including membrane blebbing, cell shrinkage, nuclear fragmentation, chromatin condensation and chromosomal DNA fragmentation. During apoptosis, integrity of the cell membrane is maintained and an inflammatory reaction does not occur. On the other hand, unspecific cytotoxic cell death, such as necrosis, is characterized by swelling of the cells, leading to disruption of the cell membrane. In vivo, this results in a local inflammatory response, as cytoplasm and organelles are released into the extracellular space and have to be eliminated by phagocytes. Early membrane toxicity can be detected by measuring the release of large intracellular proteins such as LDH, assuming that they do not pass intact cell membranes. Tumor cells exposed to KP772 for 72 h showed typical apoptotic features, whereas cell membrane integrity was not significantly reduced within 2 h, as indicated by low LDH activity in the medium, suggesting that KP772-induced cell death is mainly apoptotic.

In addition, ex vivo experiments were performed using primary myeloblasts isolated from a bone marrow aspiration of a child with AML. Even though these cells were rather insensitive to daunorubicin, they were sensitive to treatment with KP772. As toxicity to normal cells is a major problem of chemotherapy, we compared the impact of KP772 on leukemic cells with that on healthy leukocytes. Tumor cell selectivity of KP772-induced apoptosis was observed, as healthy cells were far less strongly affected.

To further characterize KP772-induced apoptosis, we investigated the pathways involved. Basically, two main signal transduction pathways lead to programmed cell death: the intrinsic and the extrinsic pathway. Both finally involve caspases, which are responsible for the characteristic morphological changes and biochemical processes of apoptosis. The extrinsic pathway is triggered by death receptors of the TNF (tumor necrosis factor) receptor superfamily, which transmit apoptotic signals into the cell upon binding of specific ligands; e.g., apoptosis induced by CD95 (Fas/APO-1) activates caspase-8, which contains an N-terminus with FADD(Fas-associated protein with death domain)-like death effector domains [2, 4]. However, an overexpression of a dominant negative FADD mutant did not impair KP772-induced apoptosis compared to mock-transfected BJAB cells, indicating independence from the CD95 receptor. The intrinsic or mitochondrial pathway, on the other hand, involves the release of cytochrome C and other pro-apoptotic factors such as Smac/DIABLO from mitochondria into the cytoplasm. As shown previously, KP772 induces a decrease of the mitochondrial membrane potential, indicating that the intrinsic pathway is involved in KP772-induced apoptosis [7].

Inactivation of apoptotic pathways plays a decisive role in the development of drug resistance. Only drugs triggering apoptosis independently of the inactivated pathway can overcome this resistance. To get an insight into the pathways involved in the case of KP772, we used cellular model systems with specific deficiencies in apoptotic signaling, in particular the pro-apoptotic factor Smac and the anti-apoptotic protein Bcl-2. Smac is a caspase-activating protein released from mitochondria during activation of the intrinsic apoptotic pathway. Transfection of smac sensitizes tumor cells to drug-induced apoptosis by enhancement of mitochondrial Bax accumulation and inhibition of Bcl-2-mediated anti-apoptotic activity [27]. As shown here, KP772-induced apoptosis is independent of Smac. Members of the Bcl-2 family play a central regulatory role in apoptosis induction and are differentially expressed in various malignancies [28]. For example, the overexpression of anti-apoptotic Bcl-2 in human melanoma cells leads to resistance to many cytostatic drugs [1]. However, KP772 is capable of overcoming this resistance and induces apoptosis in Bcl-2-overexpressing cells as efficiently as in control cells. Although cleavage of caspase-3 during KP772-induced apoptosis has been reported [7], KP772 also induces apoptosis in caspase-3-deficient multi-resistant MCF7 cells, indicating that the cytotoxic effect is partly independent of caspase-3.

By means of real-time PCR, we investigated the mRNA expression of 84 apoptosis-relevant genes during KP772-induced apoptosis. Remarkably, upregulation of the BH3-only protein Harakiri, which belongs to the Bcl-2 family, was detected. As Harakiri increases the sensitivity to apoptosis by interaction with anti-apoptotic Bcl-2 [29], KP772 might be effective in the treatment of malignancies which show drug resistance due to overexpression of Bcl-2.

Expression of ATP-binding cassette (ABC) transporters (i.e., P-glycoprotein, multidrug resistance-associated proteins) plays another important role in drug resistance. Overexpression of these transporters has been observed in many malignancies and is associated with poor response to chemotherapy [30]. In vincristine-resistant NALM-6 cells used here, significant upregulation of P-gp and additional resistance to paclitaxel and fludarabine could be detected, thereby fulfilling the criteria of multidrug resistance. In contrast, no upregulation of P-gp was found in the selectively doxorubicin-resistant cell line 7CCA derived from BJAB cells. In these resistant cell models, the effect of KP772 was even slightly superior to that in the parental cells lacking the resistance. This confirms the previously reported observation that P-gp does not interfere with KP772 and that P-gp-overexpressing multidrug-resistant cells may even show hypersensitivity to KP772 [13], but also demonstrates the capacity of overcoming P-gp-independent drug resistance.

Modern polychemotherapy combines different cytostatic drugs to utilize synergistic effects and to minimize adverse reactions. When combined with the established drugs doxorubicin, vincristine and cytarabine, KP772 showed impressive synergistic effects, which suggests KP772 as a candidate for polychemotherapy. The prerequisite of a non-overlapping toxicity profile in vivo remains to be clarified, however.

Taken together, our data demonstrate the excellent apoptosis-inducing potency of KP772 and its remarkable ability to overcome multiple drug resistances. The induction of apoptosis is accompanied by upregulation of the pro-apoptotic BH3-only protein Harakiri, explaining the activity of KP772 in cells overexpressing anti-apoptotic Bcl-2, which are usually rather insensitive to chemotherapy. As the development of drug resistances is still a major problem in clinical treatment, these findings support KP772 as a very promising drug candidate, especially for the treatment of drug-refractory malignancies.

## Supplementary Information

Below is the link to the electronic supplementary material.

Supplementary file1 (PDF 1080 kb)

## Declarations

### Availability of data and materials

The data that support the findings of this study are available from the corresponding author upon reasonable request.
