# Supplementary material for: KP772 overcomes multiple drug resistance in malignant lymphoma and leukemia cells in vitro by inducing Bcl-2-independent apoptosis and upregulation of Harakiri
Source: J Biol Inorg Chem. 2021 Oct 6;26(8):897–907. doi: 10.1007/s00775-021-01900-9 (PMC8557194; doi:10.1007/s00775-021-01900-9)
Supplement: Supplementary file 1 — Supplementary file1 (PDF 1080 kb) [file 775_2021_1900_MOESM1_ESM.pdf]

SUPPORTING INFORMATION

KP772 overcomes multiple drug resistance in malignant lymphoma and leukemia cells in vitro by inducing Bcl-2-independent apoptosis and upregulation of Harakiri

Lisa Kater, Benjamin Kater, Michael A. Jakupec, Bernhard K. Keppler, Aram Prokop

Table of content

LDH-release assay 2

Inhhibition of cancer cell proliferation 3

LDH-release assay

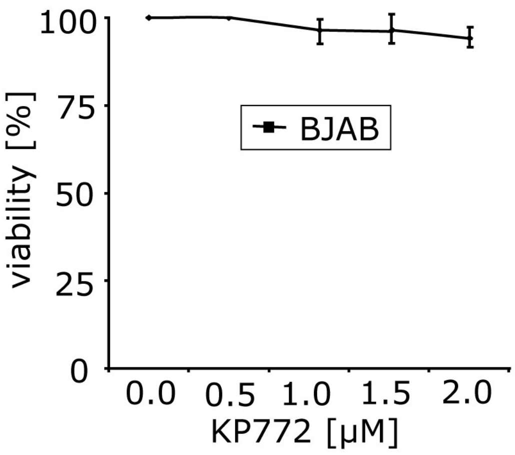

Fig. S1 Significant necrosis was excluded by the LDH-release assay after 2 h of incubation of BJAB cells with different concentrations of KP772. Some cells were left untreated as controls. Data are displayed as viability. Error bars represent standard deviations of the mean of three experiments

Inhibition of cancer cell proliferation

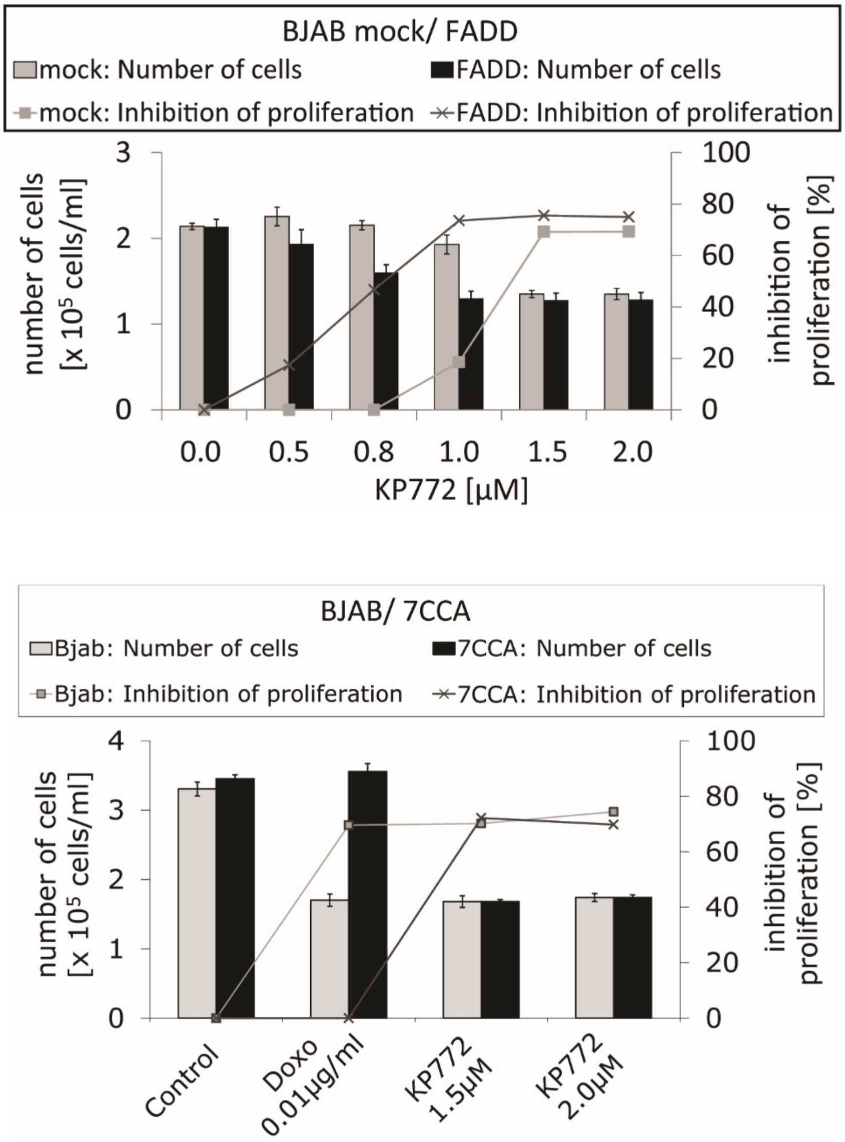

Fig. S2 Effects of 24-h exposure to KP772 in two different drug-resistant cell subclones of the Burkitt-like lymphoma cell line BJAB. Top: BJAB FADD-dn cells (overexpressing a dominant negative FADD mutant) compared to BJAB mock cells (expressing endogenous FADD only); bottom: doxorubicin-resistant (7CCA) and normal BJAB cells. Values are mean % of control ± SD (*n* = 3).

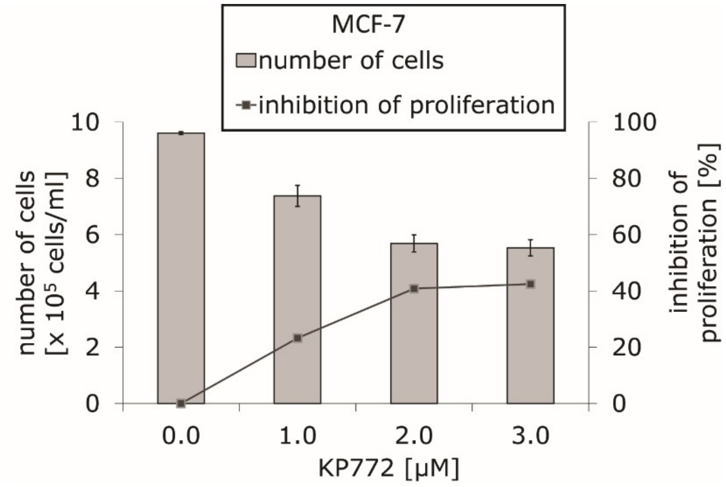

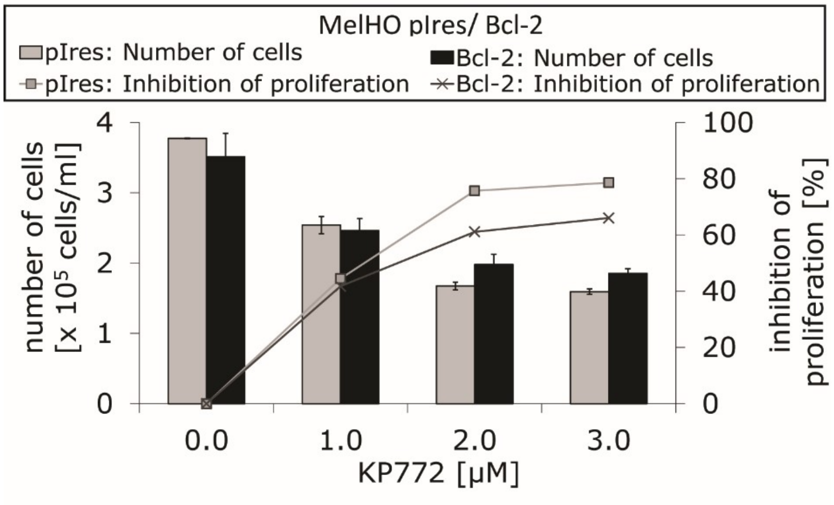

Fig. S3 Effects of 24-h exposure to KP772 in cancer cell lines with drug resistances. Top: breast cancer cell line MCF-7; bottom: melanoma cell line MelHO Bcl-2 (overexpressing anti-apoptotic Bcl-2 protein) in comparison to MelHO pIres. Values are mean % of control ± SD (n = 3).

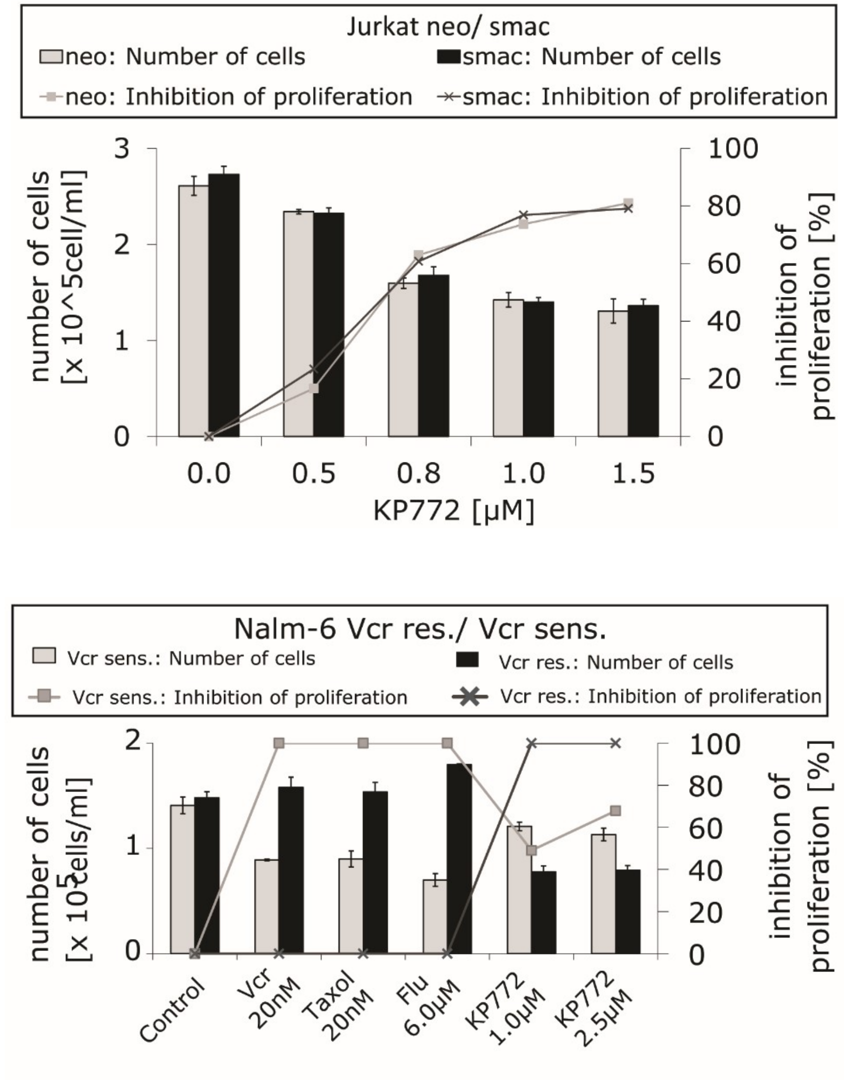

Fig. S4 Effects of 24-h exposure to KP772 in cancer cell lines with drug resistances. Top: SMAC protein overexpressing human T-cell leukemia cell line Jurkat smac compared to Jurkat neo cells; bottom: vincristine-resistant leukemia cells (Nalm-6/Vcr) and the corresponding nonresistant cells (Nalm-6); the vincristine-resistant cells were also resistant to other common drugs, as shown for paclitaxel (Taxol, 20 nM) and fludarabine (Flu, 6 µM); vincristine was used in concentrations up to 20 nM. Values are mean % of control ± SD (n = 3).

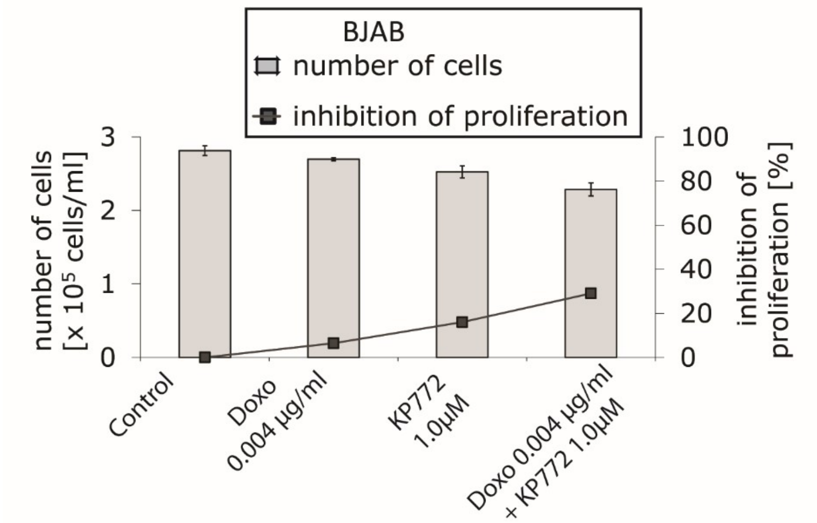

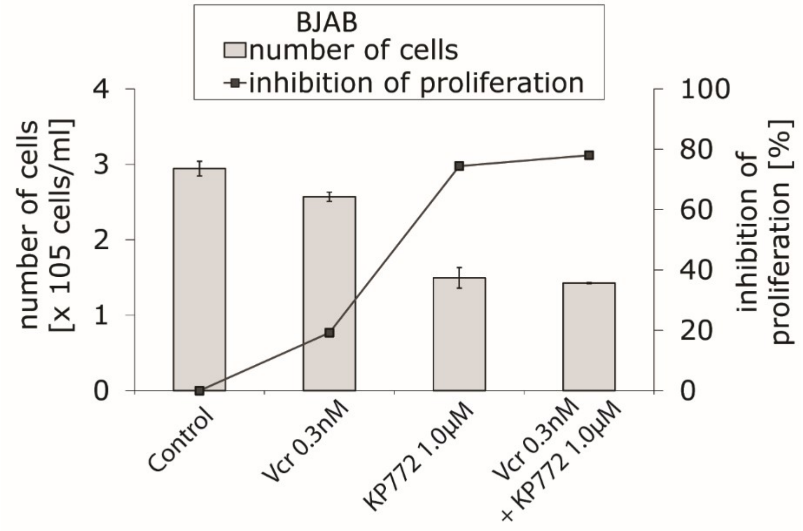

Fig. S5 Effects of 24-h exposure of BJAB cells to KP772 (1 µM) or other chemotherapeutic drug alone and a combination thereof. Top: doxorubicin (Doxo, 0.004 µg/ml); bottom: vincristine (Vcr, 0.3 nM). Values are mean % of control ± SD (*n* = 3).

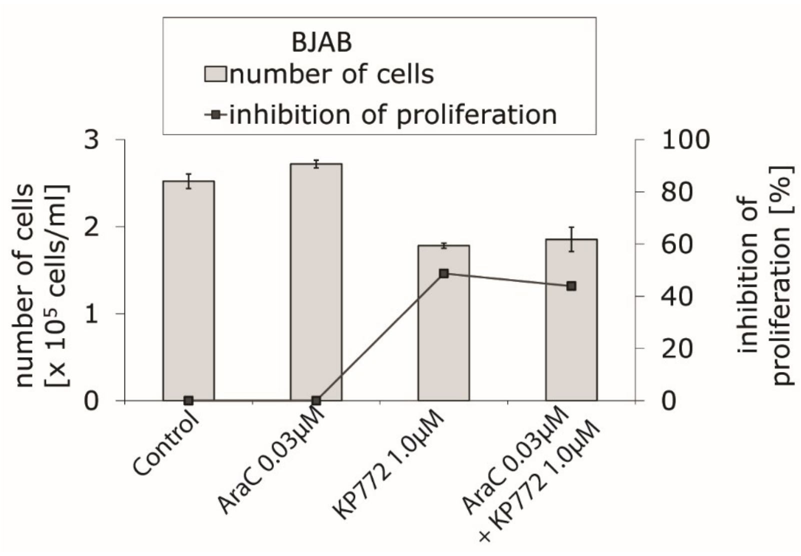

Fig. S6 Effects of 24-h exposure of BJAB cells to KP772 (1 μM) or cytarabine (AraC, 0.3 μM) alone and their combination. Values are mean % of control ± SD (*n* = 3).
